# Exploring the Awareness and Application of Artificial Intelligence in Dentistry: A Cross-Sectional Analysis of Dentists and Patients

**DOI:** 10.7759/cureus.82947

**Published:** 2025-04-24

**Authors:** Jai krishna Grosu, Sreenivas Nagarakanti, Tejasri Gudur, Rishitha G, Charitha Neravati, Rithika Madireddygari

**Affiliations:** 1 Periodontology, Narayana Dental College and Hospital, Nellore, IND

**Keywords:** artificial intelligence (ai), cross-sectional study, dental practice, dentistry, patients

## Abstract

Background

Artificial intelligence (AI) is rapidly transforming healthcare, including the field of dentistry, by enhancing diagnostic accuracy, treatment planning, and patient management. However, the perception and extent of AI utilization among dentists and patients remain largely unexplored. This study aims to assess the awareness, perception, and application of AI in dentistry among both practitioners and patients.

Methodology

A cross-sectional survey was conducted among dentists and patients to evaluate their familiarity, perception, and utilization of AI-powered tools in dental practice. A structured questionnaire was used to collect data on AI awareness, perceived benefits and concerns, and its application in diagnosis, treatment planning, and patient engagement. Statistical analyses, including chi-square tests, were performed to identify associations between demographic variables and AI perception.

Results

The findings revealed that a significant proportion of dentists (60%) were aware of AI applications in dentistry, with higher familiarity among those with advanced education and professional experience. However, actual utilization remained limited, primarily due to concerns about reliability, cost, and ethical implications. Patients (60%) demonstrated varied levels of awareness, with younger and more educated individuals expressing a greater interest in AI-assisted dental care.

Conclusions

AI is perceived as beneficial in enhancing diagnostic accuracy and treatment efficiency, yet skepticism persists regarding its potential to replace human expertise. While AI is acknowledged as a valuable tool in dentistry, its adoption remains in the early stages. Increased education and training initiatives are necessary to bridge the knowledge gap and improve acceptance among both dentists and patients. Addressing ethical concerns and integrating AI seamlessly into dental practice could enhance its utilization, ultimately improving patient outcomes and clinical efficiency.

## Introduction

Artificial intelligence (AI) has rapidly integrated into various healthcare sectors, offering innovative solutions that enhance diagnostic accuracy, treatment planning, and patient management [1,2]. In dentistry, AI applications have demonstrated significant potential in improving clinical outcomes and operational efficiencies. For instance, AI algorithms have been employed to assist in the detection of dental caries, periodontal diseases, and even oral cancers, thereby aiding clinicians in early intervention and improving treatment outcomes [3,4].

The integration of AI into dental practices encompasses several key areas, including diagnosis, treatment planning, image analysis, patient management, and personalized care [1]. AI has been utilized for the identification of periapical lesions and root anatomy in endodontics, as well as for the diagnosis of periodontitis [3]. Additionally, AI has been applied in clinical decision-making, aiding in detecting disease and predicting patterns based on existing datasets [5]. Despite these advancements, the perceptions and attitudes of both dental professionals and patients toward AI integration remain varied [3].

Studies indicate that while a majority of dental professionals acknowledge the potential benefits of AI, there is a spectrum of awareness and readiness to adopt these technologies [6-9]. For example, a study assessing the knowledge, attitude, and perception regarding AI among periodontists revealed that most do not currently use AI for diagnostic or research purposes, with 49% having a view that AI does not have better diagnostic accuracy than periodontists [10]. Similarly, a study on the attitudes and perceptions of Australian dentists and dental students found that while 54.8% were aware of dental AI applications, 70.3% could not name a specific AI software [11].

Patients, on the other hand, have shown a generally positive attitude toward AI in dentistry. A study exploring patients’ perspectives found that major expected advantages of AI use in dentistry were improved diagnostic confidence (60.8%), time reduction (48.3%), and more personalized and evidence-based disease management (43.0%) [12]. Understanding these perceptions is crucial, as they can influence the acceptance and successful implementation of AI-driven solutions in dental care [4].

Despite the potential of AI in dentistry, challenges such as data privacy, algorithm bias, and regulatory considerations persist. Addressing these issues requires collaborative efforts between dental professionals, AI experts, and policymakers to develop robust frameworks that ensure the responsible and ethical implementation of AI in dentistry [13,14]. This understanding will guide the development and integration of AI technologies that are both effective and aligned with the expectations and needs of end-users [4].

Hence, this study aims to explore the awareness, knowledge, attitudes, and practical application of AI in dentistry from both the perspectives of dental professionals and patients.

## Materials and methods

Study design

A descriptive, cross-sectional study was performed to assess the perception and utilization of AI in Nellore district, Andhra Pradesh, specifically targeting dentists (BDS/MDS), dental interns, and postgraduate dental students practicing or studying within the district as well as patients seeking or visiting for dental care at Narayana Dental College and Hospital, Nellore. The study relied on primary data collected through a structured questionnaire survey administered to the target population.

Inclusion and exclusion criteria

Dentists (BDS/MDS), interns, and dental postgraduates working or studying in Nellore district, Andhra Pradesh, and patients visiting Narayana Dental College and Hospital, Nellore, for dental care were considered for inclusion in the study. Individuals capable of accessing computers, mobile devices, and the internet were included. Respondents provided informed consent to participate in the study. Participants who did not provide informed consent and who were unable to comprehend the survey questionnaire were excluded.

Ethical clearance

Ethical approval for the study was obtained from the Institutional Ethics Committee, Narayana Dental College and Hospital, Nellore, on August 6, 2024 (approval number: IEC/NDCH/2024/P-65).

Sampling method and sample size

A convenience sampling method was employed over two months to recruit participants. Investigators personally approached the participants with a validated, structured questionnaire and collected the relevant information. The questionnaire was divided into three parts, including demographic details, familiarity with AI in general and medical, application in dentistry, and for dentists, additional questions regarding the barriers of AI incorporation into their practice were asked.

The content validity of the questionnaire was assessed using the Davis criteria [15,16], and two experts in dental research assessed the questionnaire, with an item content validity index of 1.0 and a scale content validity index of 0.9 [17]. The reliability of the questionnaire was evaluated by conducting a pilot study on a subset of the population before the main study. Cronbach’s alpha was calculated to determine the internal consistency of the questionnaire.

Data analysis

Data were analyzed using a statistical software program (GNU PSPP 2.0.1). Descriptive statistics were employed to gain an initial understanding of the dataset. Specifically, the chi-square test was employed to assess the significant relationship between the variables. A p-value of less than 0.001 was considered to be significant.

## Results

Dentists

Table 1 summarizes the demographic characteristics and AI awareness of participants. Age was significantly associated with AI awareness (p < 0.001); younger participants (aged 20-39 years) universally reported awareness, while those aged over 60 years had no awareness. Intermediate awareness was observed in participants aged 40-49 (51.3%) (n = 20) and 50-59 years (43.8%) (n = 21). AI awareness did not significantly differ between genders, with both male and female respondents being equally aware (60%). Educational qualifications significantly impacted awareness (p < 0.001), with interns and postgraduates showing complete awareness (100%) (n = 26) and BDS-qualified individuals showing the lowest (42.3%) (n = 44). Type of service (p = 0.001) and place of practice (p = 0.003) also significantly influenced awareness, with the highest rates observed in private practitioners (73.0%) (n = 73) and urban practitioners (71.7%) (n = 71), respectively. Additionally, AI awareness significantly decreased with increased years in practice and patient load per day (both p < 0.001).

**Table 1:** Demographic data. Chi-square test. *: P-values <0.001 were considered statistically significant. AI: artificial intelligence

Parameters	Aware of AI - yes	Aware of AI - no	P-value	Chi-square value
Age group (years)				
20–29	44 (100.0%)	0 (0.0%)	<0.001*	110.18
30–39	35 (100.0%)	0 (0.0%)		
40–49	20 (51.3%)	19 (48.7%)		
50–59	21 (43.8%)	27 (56.3%)		
>60	0 (0.0%)	34 (100.0%)		
Gender				
Male	60 (60.0%)	40 (40.0%)	1	0
Female	60 (60.0%)	40 (40.0%)		
Educational qualification				
Intern	8 (100.0%)	0 (0.0%)	<0.001*	37.78
Postgraduate	26 (100.0%)	0 (0.0%)		
BDS	44 (42.3%)	60 (57.7%)		
MDS	42 (67.7%)	20 (32.3%)		
Type of service				
Public	18 (42.9%)	24 (57.1%)	<0.001*	14.60
Private	73 (73.0%)	27 (27.0%)		
Teaching institute	29 (50.0%)	29 (50.0%)		
Place of practice				
Urban	71 (71.7%)	28 (28.3%)	0.003	11.34
Rural	20 (46.5%)	23 (53.5%)		
Teaching institute	29 (50.0%)	29 (50.0%)		
Years in practice				
0–2	17 (100.0%)	0 (0.0%)	<0.001*	68.69
2–5	24 (100.0%)	0 (0.0%)		
5–10	27 (100.0%)	0 (0.0%)		
>10	52 (39.4%)	80 (60.6%)		
Average patients per day				
<10	33 (100.0%)	0 (0.0%)	<0.001*	27.47
11–30	61 (55.0%)	50 (45.0%)		
31–50	26 (46.4%)	30 (53.6%)		

Table 2 presents participant familiarity with AI. Significant associations (p < 0.001) were observed between AI awareness and previous AI usage, familiarity with AI devices, medical use, and dentistry-specific applications. All participants who previously used AI outside medical contexts had familiarity with AI devices or utilized AI medically (100%) (n = 120). Conversely, no awareness was reported by those lacking such experiences. Those unfamiliar with AI in medical contexts (47%) (n = 71) were reported to be aware of AI compared to the awareness among those with even slight familiarity (100%) (n = 25). Participants who had not used AI in medical contexts showed moderate awareness (53.2%) (n = 91), whereas 25.9% (n = 28) of participants not using AI in dentistry reported AI awareness.

**Table 2:** Familiarity with AI. Chi-square test. *: P-values <0.001 were considered statistically significant. AI: artificial intelligence

Parameters	Aware of AI - yes	Aware of AI - no	P-value	Chi-square value
Used AI for purposes other than medical				
Yes	120 (100.0%)	0 (0.0%)	<0.001*	200
No	0 (0.0%)	80 (100.0%)		
Familiarity with AI devices				
Not familiar at all	0 (0.0%)	80 (100.0%)	<0.001*	200
Slightly familiar	32 (100.0%)	0 (0.0%)		
Moderately experienced	49 (100.0%)	0 (0.0%)		
Very experienced	28 (100.0%)	0 (0.0%)		
Extremely experienced	11 (100.0%)	0 (0.0%)		
Used AI in medicine				
Yes	29 (100.0%)	0 (0.0%)	<0.001*	22.61
No	91 (53.2%)	80 (46.8%)		
Familiarity with AI in medicine				
Not familiar at all	71 (47.0%)	80 (53.0%)	<0.001*	43.27
Slightly familiar	25 (100.0%)	0 (0.0%)		
Moderately experienced	13 (100.0%)	0 (0.0%)		
Very experienced	4 (100.0%)	0 (0.0%)		
Extremely experienced	7 (100.0%)	0 (0.0%)		
AI in dentistry				
Yes	92 (100.0%)	0 (0.0%)	<0.001*	113.58
No	28 (25.9%)	80 (74.1%)		

Table 3 outlines the application of AI across different domains of dentistry. The most common use of AI reported by the participants was for appointments (46.7%) (n = 43). Treatment planning was reported by participants (37.0%) (n = 34) following AI use for appointments. Lower levels of AI usage were reported for treatment procedures (28.3%) (n = 26), clarifying doubts (26.1%) (n = 24), prognosis (25.0%) (n = 23), and diagnosis (22.8%) (n = 21). Notably, the majority of respondents indicated limited or no use of AI for diagnostic and prognostic purposes, with non-usage rates of 77.2% (n = 71) and 75.0% (n = 69), respectively.

**Table 3:** Use of AI in dentistry. AI: artificial intelligence

Parameters	Yes	No
Appointments	43 (46.7%)	49 (53.3%)
Diagnosis	21 (22.8%)	71 (77.2%)
Prognosis	23 (25.0%)	69 (75.0%)
Treatment plan	34 (37.0%)	58 (63.0%)
Treatment procedure	26 (28.3%)	66 (71.7%)
Clarifying doubts	24 (26.1%)	68 (73.9%)

Table 4 highlights the perceived barriers to integrating AI into dentistry. Significant barriers (p < 0.001) included lack of awareness, trained personnel, technical resources, curricular integration, perceived limited future use, and trustworthiness. A larger proportion of non-users identified a lack of awareness (68.6%), limited future applicability (71.4%), and trustworthiness concerns (73.6%) compared to AI users. Conversely, higher proportions of current AI users acknowledged a lack of trained personnel (84.6%), technical resources (76.5%), and curricular integration issues (79.2%), indicating recognition of practical limitations. Cost was not identified as a significant barrier, with comparable proportions reporting this issue among users and non-users (p = 0.788).

**Table 4:** Barriers of AI integration in dentistry. Chi-square test. *: P-values <0.001 were considered statistically significant. AI: artificial intelligence

Parameters	AI in dentistry - yes	AI in dentistry - no	P-value	Chi-square value
Lack of awareness				
Yes	43 (31.4%)	94 (68.6%)	<0.001*	37.39
No	49 (77.8%)	14 (22.2%)		
Lack of training personnel				
Yes	22 (84.6%)	4 (15.4%)	<0.001*	17.94
No	70 (40.2%)	104 (59.8%)		
Lack of technical resources				
Yes	26 (76.5%)	8 (23.5%)	<0.001*	15.31
No	66 (39.8%)	100 (60.2%)		
Non-essentiality in the curriculum				
Yes	19 (79.2%)	5 (20.8%)	<0.001*	12.08
No	73 (41.5%)	103 (58.5%)		
Expensive				
Yes	19 (44.2%)	24 (55.8%)	0.788	0.07
No	73 (46.5%)	84 (53.5%)		
Limited future use				
Yes	36 (28.6%)	90 (71.4%)	<0.001*	41.64
No	56 (75.7%)	18 (24.3%)		
Not trustworthy				
Yes	34 (26.4%)	95 (73.6%)	<0.001*	56.54
No	58 (81.7%)	13 (18.3%)		

Patients

Table 5 summarizes demographic characteristics and AI awareness among patients. Significant associations were observed between AI awareness and age, educational level, employment status, and annual income (all p < 0.001). Younger participants (aged 20-29 years) universally reported AI awareness (100%), with awareness progressively decreasing among older age groups, reaching zero in participants aged over 60 years. Educational attainment was positively correlated with AI awareness, with the highest among college students (100%) and the lowest among high school graduates (11.3%). Employment status also influenced awareness, with full awareness reported among part-time employees and students, moderate awareness among full-time employed respondents (55.7%), lower awareness among unemployed respondents (35.7%), and none among retirees. Additionally, higher annual income correlated with greater AI awareness, peaking in individuals earning above INR 10 lakhs per annum (73.5%), and lowest in those earning below INR 1.5 lakhs per annum (32.6%). Gender did not significantly affect AI awareness (p = 0.92).

**Table 5:** Demographic data. Chi-square test. *: P-values <0.001 were considered statistically significant. AI: artificial intelligence

Parameters	Aware of AI - yes	Aware of AI - no	P-value	Chi-square value
Age group (years)				
20–29	51 (100.0%)	0 (0.0%)	<0.001*	107.37
30–39	35 (76.1%)	11 (23.9%)		
40–49	17 (50.0%)	17 (50.0%)		
50–59	17 (44.7%)	21 (55.3%)		
>60	0 (0.0%)	45 (100.0%)		
Gender				
Male	63 (55.8%)	50 (44.2%)	0.92	0.01
Female	57 (56.4%)	44 (43.6%)		
Education level				
Less than high school	8 (44.4%)	10 (55.6%)	<0.001*	100.14
High school graduate	8 (11.3%)	63 (88.7%)		
College student	17 (100.0%)	0 (0.0%)		
Bachelor’s degree	59 (76.6%)	18 (23.4%)		
Master’s degree	28 (90.3%)	3 (9.7%)		
Employment status				
Full time	73 (55.7%)	58 (44.3%)	<0.001*	56.58
Part time	9 (100.0%)	0 (0.0%)		
Unemployed	10 (35.7%)	18 (64.3%)		
Retired	0 (0.0%)	18 (100.0%)		
Student	28 (100.0%)	0 (0.0%)		
Annual income				
INR lakhs per annum	14 (32.6%)	29 (67.4%)	<0.001*	18.54
INR 1.5 to 3 lakhs per annum	31 (50.0%)	31 (50.0%)		
INR 3 to 10 lakhs per annum	39 (65.0%)	21 (35.0%)		
>INR 10 lakhs per annum	36 (73.5%)	13 (26.5%)		

Table 6 highlights participant familiarity with AI in various contexts. Significant associations (all p < 0.001) were observed between AI awareness and previous use of AI for non-medical purposes, familiarity with AI technology, use of AI-powered health services, familiarity with AI in healthcare, and AI use in dentistry. All participants who had experience with AI for non-medical purposes, used AI-powered health services, or had slight-to-high familiarity with AI reported complete awareness (100%) (n = 86). Conversely, lower awareness rates were noted among those without prior AI experience or familiarity. Additionally, 93.5% (n = 58) of respondents using AI in dentistry reported general AI awareness compared to only 40.8% (n = 62) among non-users.

**Table 6:** Familiarity with AI. Chi-square test. *: P-values <0.001 were considered statistically significant. AI: artificial intelligence

Parameters	Aware of AI - yes	Aware of AI - no	P-value	Chi-square value
Used AI for purposes other than medical				
Yes	86 (100.0%)	0 (0.0%)	<0.001*	112.63
No	34 (26.6%)	94 (73.4%)		
Familiarity with AI				
Not familiar at all	37 (28.2%)	94 (71.8%)	<0.001*	106.21
Slightly familiar	29 (100.0%)	0 (0.0%)		
Moderately experienced	35 (100.0%)	0 (0.0%)		
Very experienced	19 (100.0%)	0 (0.0%)		
Used AI-powered health services				
Yes	48 (100.0%)	0 (0.0%)	<0.001*	48.47
No	72 (43.4%)	94 (56.6%)		
Familiarity with AI in healthcare				
Not familiar at all	67 (41.6%)	94 (58.4%)	<0.001*	55.18
Slightly familiar	32 (100.0%)	0 (0.0%)		
Moderately experienced	21 (100.0%)	0 (0.0%)		
AI in dentistry				
Yes	58 (93.5%)	4 (6.5%)	<0.001*	47.65
No	62 (40.8%)	90 (59.2%)		

Table 7 outlines the application of AI across various dentistry-related tasks. The most frequently reported use of AI was in estimating treatment costs (74.1%) (n = 43), followed by performing treatment procedures (69.0%) (n = 40) and clarifying patient doubts (65.5%) (n = 38). Moderate use was reported for diagnosis (60.3%) (n = 35) and treatment planning (58.6%) (n = 34). The least common application of AI was for managing appointments (51.7%) (n = 30), where nearly half of the respondents reported no AI use (48.3%) (n = 28).

**Table 7:** Use of AI in dentistry. AI: artificial intelligence

Parameters	Yes	No
Appointments	30 (51.7%)	28 (48.3%)
Diagnosis	35 (60.3%)	23 (39.7%)
Treatment plan	34 (58.6%)	24 (41.4%)
Clarifying doubts	38 (65.5%)	20 (34.5%)
Treatment procedure	40 (69.0%)	18 (31.0%)
Treatment cost	43 (74.1%)	15 (25.9%)

## Discussion

AI is increasingly transforming the landscape of dental practice, offering significant potential in enhancing diagnostic precision, treatment planning, clinical workflow efficiency, and patient management [18]. The present study demonstrates that demographic variables play a pivotal role in influencing awareness and receptivity toward AI in dentistry. Notably, younger individuals, those with higher educational qualifications, and participants belonging to higher-income groups exhibited significantly greater awareness of AI technologies. This could be because individuals with higher education and income levels typically demonstrate greater AI awareness, reflecting broader exposure to AI curricula, enhanced digital literacy, and improved access to technological resources and information. Younger individuals adopt technologies more readily due to active engagement with digital platforms and cultural trends involving AI. Additionally, higher-income professionals frequently encounter AI in their occupations, further increasing familiarity and understanding of its applications.

To bridge the awareness and utilization gap, targeted educational strategies are essential. Integrating AI-based content into undergraduate and postgraduate dental curricula can build foundational knowledge and practical competence among future dental practitioners [19-21]. Furthermore, continuous professional development programs must be prioritized to update practicing clinicians on advancements in AI applications and to facilitate lifelong learning [21-23]. In parallel, collaborations among dental institutions, AI experts, software developers, and policy-makers are necessary to formulate standardized frameworks, ethical guidelines, and implementation protocols tailored for AI adoption in dental settings [24].

The study findings reveal that prior exposure to AI, whether through general technological use or medical applications, was strongly associated with increased awareness and positive perception. This underscores the importance of experiential learning in building confidence and familiarity with AI tools. However, despite a generally favorable outlook, the actual application of AI in clinical dental practice remains limited. The most commonly reported uses among participants included cost estimation, radiographic interpretation, diagnostic assistance, and treatment planning, areas that align closely with those identified in current research as early focal points for AI integration in dentistry [8].

Numerous barriers were identified that hinder broader AI adoption. These include insufficient integration of AI principles and applications in dental education, limited interdisciplinary collaboration between dental professionals and AI technologists, ethical concerns, and the lack of structured hands-on training. A considerable proportion of respondents reported that inadequate technical infrastructure, absence of trained support personnel, and lack of exposure to AI tools during formal education posed significant challenges [25,26]. Interestingly, contrary to common assumptions, financial limitations were not reported as a predominant barrier. This suggests that infrastructural, pedagogical, and experiential shortcomings outweigh cost concerns in influencing AI adoption.

Trust-related issues also emerged as significant deterrents. Participants unfamiliar with AI expressed concerns regarding data security, reliability of machine-generated outputs, and lack of transparency in algorithmic decision-making processes. Addressing these concerns is imperative to promote trust and acceptance. Establishing robust regulatory frameworks that prioritize ethical AI practices, such as ensuring algorithmic transparency, protecting patient privacy, and incorporating clinician oversight, will be instrumental in overcoming skepticism and fostering wider AI integration in clinical dentistry [27].

To mitigate these barriers, several implementation strategies have been recommended. International literature supports the use of demonstrative case studies that highlight the clinical benefits and efficiency gains associated with AI integration as a means to foster acceptance among dental professionals. Embedding structured evaluation and feedback mechanisms within AI deployment protocols has also been shown to enhance implementation success [27-30]. Furthermore, institutional leadership and effective communication are critical enablers. Encouragingly, the involvement of local clinical champions, dentists who advocate for AI within their institutions, has been identified as a key factor in overcoming resistance. These individuals can facilitate peer-to-peer knowledge transfer, address concerns, and lead by example, thereby serving as catalysts for successful AI adoption in dental settings [31,32].

The successful implementation of AI in dentistry hinges on a multifaceted approach that includes addressing educational deficits, enhancing hands-on exposure, fostering interdisciplinary collaboration, and establishing clear regulatory and ethical frameworks. Increasing AI literacy among both emerging and practicing dental professionals through curriculum reform and continuous training is paramount. Additionally, building trust in AI systems through transparent communication, regulatory safeguards, and clinician-led advocacy is essential to promoting widespread adoption.

Future research should focus on longitudinal studies evaluating the clinical effectiveness, patient outcomes, cost-efficiency, and user satisfaction associated with AI-based interventions. Moreover, incorporating patient perspectives into implementation strategies will ensure that AI adoption remains patient-centered and ethically grounded. As AI technology continues to evolve, its integration into dentistry must be guided by evidence-based, inclusive, and strategically coordinated efforts to ensure its sustainable and effective use in clinical practice.

This study is subject to certain limitations. The use of a convenience sampling method may introduce selection bias and limit the representativeness of the sample. Furthermore, the study was geographically restricted, which may affect the generalizability of the findings to broader populations. Additionally, the reliance on self-reported data introduces the potential for response bias, as participants’ perceptions may not accurately reflect actual knowledge or clinical behavior.

## Conclusions

While AI holds significant promise for transforming dental practice, realizing its full potential requires concerted efforts to overcome existing barriers. By prioritizing education, fostering trust, and promoting collaborative initiatives, the dental community can harness AI’s capabilities to enhance patient care and advance the profession.​
